# An app-based training for adolescents with problematic digital-media use and their parents (Res@t digital): protocol for a cluster-randomized clinical trial

**DOI:** 10.3389/fpsyt.2023.1245536

**Published:** 2024-01-24

**Authors:** Kerstin Paschke, Silke Diestelkamp, Antonia Zapf, Katharina Busch, Nicolas Arnaud, Alexander Prehn-Kristensen, Olaf Reis, Maria Stark, Jan-Ole Cloes, Anna-Lena Schulz, Hannah Brauer, Thomas Krömer, Rainer Thomasius, the Res@t Consortium

**Affiliations:** ^1^German Center for Addiction Research in Childhood and Adolescence (DZSKJ), University Medical Center Hamburg-Eppendorf (UKE), Hamburg, Germany; ^2^Institute of Medical Biometry and Epidemiology (IMBE), University Medical Center Hamburg-Eppendorf (UKE), Hamburg, Germany; ^3^Institute of Child and Adolescent Psychiatry, Center for Integrative Psychiatry, School of Medicine, Christian-Albrecht University Kiel, Kiel, Germany; ^4^Department for Child and Adolescent Psychiatry and Neurology, Rostock University Medical Center, Rostock, Germany; ^5^Collaborative Practice for Child and Adolescent Psychiatry, Psychotherapy and Psychosomatics, Hamburg, Germany

**Keywords:** digital media-use disorders, e-health, digital intervention, cognitive-behavioral therapy, adolescents, parents

## Abstract

**Background:**

Digital media-use disorders (DMUD) in adolescents are a rising phenomenon associated with psychological distress, comorbid mental disorders, and high burden on affected families. Since the ICD-11 introduced criteria for gaming disorder, these can now be transferred to describe additional DMUD associated with social media platforms and streaming services. Most evidence for effective treatments comes from cognitive-behavioral therapy (CBT). However, interventions based on theoretical models for adolescents and their parents are widely missing, leading to a significant clinical gap.

**Methods:**

Res@t digital (Resource-Strengthening Training for Adolescents with Problematic Digital-Media Use and their Parents) is the app-based translation of the first model-based digital intervention for adolescents with DMUD and their parents based on CBT. It comprises separate but content-related modules for adolescents (Res@t–A) and parents (Res@t–P), applying multimodal techniques. The effectiveness of Res@t will be evaluated within a multicenter cluster-randomized controlled evaluator-blinded pre–post follow-up trial with the waitlist control group (CG). In addition to the Res@t program in the intervention group, both groups will receive treatment as usual within primary child and adolescent psychiatric/psychotherapeutic healthcare. The primary outcome addresses DMUD symptom reduction after 10 weeks. Secondary outcomes are related to a reduction in psychological and family-related problems and an increase in parental self-efficacy. All outcomes will be assessed using standardized self-report measures. A total of 1,334 participating adolescent–parent dyads from a large clinical network throughout Germany are planned to be included in the primary analyses based on an intention-to-treat approach, applying linear mixed models.

**Discussion:**

Assuming superiority of Res@t over the control condition, the intervention has the potential to provide evidence-based treatment for a significant number of help-seeking families, supporting local healthcare structures and resources. It is a promising program for practicable implementation and flexible use in different settings.

**Clinical trial registration:**

https://drks.de, DRKS00031043.

## 1 Introduction

The digital entertainment and media market industries belong to the fastest-growing businesses in the world (1). In 2022, revenue from digital games amounted to 184 billion U.S. dollars with mobile games accounting for 50% of the global gaming market (2). During the last decades, improved design mechanisms and broad availability led to increased usage frequency and duration worldwide over all age groups (3). During the COVID-19 pandemic, this development was fostered by contact restrictions, quarantines, and the closure of schools and leisure facilities, especially for children and adolescents (4–6). The majority of German children and adolescents use digital media on a regular basis from several times a week to daily, including social media (SM, more than 90%), video streaming (*VS*) services (more than 80%), and digital games (more than 70%) (7).

For some users, frequent digital media use as a leisure activity can turn into problematic patterns, resulting in severe negative personal, family, social, educational, and work-related sequelae with significant subsequent costs to healthcare and economic systems (8). Recent meta-analyses estimated the worldwide prevalence of problematic gaming between 3.1 and 3.3% (9, 10) and problematic SM use at 5% (11) with a peak during adolescence. Much less research has been conducted on problematic *VS*, although similar addiction-promoting mechanisms are applied to increase user bonding (12). Most associated research has focused on binge-watching, i.e., the consumption of several (television) series episodes in a row (13, 14). However, the series resembles only a small subentity of *VS* services. A representative study on German adolescents estimated the prevalence of pathologic *VS* at 4.7% in frequent (at least weekly) *VS* users (15).

Adolescents are particularly at risk for behavioral addictions including problematic gaming due to neuronal remodeling processes with a mature reward system, on the one hand, and a cognitive-control system that is still in development on the other hand (16, 17).

As the first behavioral addiction purely associated with digital media, problematic (online or offline) gaming has been included in the current (11th) version of the International Classification of Diseases (ICD-11) as *Gaming Disorder* (GD, 6C51) (18). Four criteria have been defined for diagnosing GD which must usually be present for the past 12 months: loss of control over the temporal and situational use of digital games, increased prioritization of digital gaming over alternative activities, continued or increased gaming despite negative consequences, and the occurrence of significant impairments in important areas of functioning. At the current state, other digital-media-related disorders (DMUD), such as *Social Media Use Disorder* (SMUD) or *Video Streaming Disorder* (VSD), can be classified under the umbrella *Other specific disorders due to addictive behaviors* (GC5Y), applying comparable criteria as for GD (19). Affected individuals who do not (yet) fulfill all criteria of addiction can be classified under *Hazardous Gaming* (HG, QE22) or *problems with other specified health-related behaviors* (QE2Y). The latter can include Hazardous Social Media Use (HSMU) and Hazardous Video Streaming (HVS). Disordered or hazardous usage patterns can be summarized as Problematic Gaming (PG), Problematic Social Media Use, or Problematic Video Streaming (PVS).

The etiology of DMUD can, e.g., be described by the *Triad Model of Addiction* by Kielholz & Ladewig (20). This biopsychosocial model describes an interaction of personal, social, and agent-related factors. It employs mechanisms of learning, stress management (21), and information processing and can be comprehensively applied to DRMD in adolescents. For more details, please refer to the study by Paschke et al. (22).

If DMUD remains untreated, there will be a high risk of chronicity with severe psychological consequences, repeated hospitalizations, and failure to fulfill necessary developmental tasks (e.g., no school qualifications, no education) with negative effects on the mental and physical health of the affected person and high costs for society. The best effects on symptom reduction have been shown for cognitive-behavioral therapy (CBT) (23). However, model-based and evidence-based treatment programs, addressing adolescents and their parents, are currently not available (24–26).

### 1.1 Aim of the study and hypotheses

The present study aims to evaluate the effectiveness of a new model-based standardized online intervention program, the Resource-Strengthening Training for Adolescents with Problematic Digital-Media Use, and their Parents (Res@t digital) in an outpatient setting. The Res@t digital intervention group (IG) will be compared with a waiting control group (CG). The IG will receive the Res@t digital intervention, and both groups will receive treatment as usual (TAU) during the study period. The waitlist CG will be offered access to the intervention after individual data collections are completed. It is hypothesized that Res@t digital + TAU is superior to TAU only and will lead to a decrease in adolescent DMUD (i.e., GD, SMUD, or VSD) symptoms. In the case of an adolescent being affected by more than one DMUD or hazardous use pattern, the clinician will identify the most prominent phenomenon that will be focused on within the targeted intervention. Moreover, Res@t digital will reduce mental-health-related problems in adolescents and increase parental self-efficacy.

There are three primary hypotheses structured hierarchically which address the one most prominent DMUD or hazardous use pattern in the individual adolescent identified by the clinician (i.e., PG, PSMU, or PVS). This is referred to the term “specific” DMUD:

First primary hypothesis: Res@t + TAU reduces symptoms of specific DMUD in adolescents compared with TAU, measured as change from screening to post-intervention. Second primary hypothesis: Res@t + TAU reduces symptoms of specific DMUD in adolescents assessed by their parents compared with TAU, measured as change from screening to post-intervention. Third primary hypothesis: Res@t + TAU reduces symptoms of specific DMUD in adolescents assessed by their treating clinicians compared with TAU, measured as change from screening to post-intervention.

To the best of our knowledge, although positive effects of online interventions have been repeatedly suggested to fill substantial treatment gaps for mental health issues in adults (27–29) and adolescents (30–32) including addictive behavior (33–35), no comparable intervention for adolescents with DMUD and their parents is available yet. Therefore, Res@t strives to close a significant void.

## 2 Methods and analysis

### 2.1 Study design

The current study is a multicenter, prospective, cluster randomized-controlled, observer-blind clinical trial on the effectiveness of Res@t regarding symptom reduction in adolescents with DMUD. A two-arm study design will be applied with an IG and a waitlist CG with pre–post follow-up assessments. All patients will receive surveillance by a child and adolescent psychiatrist and psychotherapist who offers TAU in regular treatment parallel to the study.

### 2.2 Study sample and setting

Adolescents aged 10 to 19 years (based on the WHO definition of adolescence) and their respective parents, i.e., caregivers, will be recruited for the study by their child and adolescent psychiatrists and/or psychotherapists. Recruitment sites will include 10 outpatient departments of participating clinics (five university hospitals, five large care clinics) and approximately 40 practices throughout Germany (coordinated via the Professional Association for Child and Adolescent Psychiatry, Psychosomatics, and Psychotherapy in Germany e.V., BKJPP).

All eligible recruitment sites will be assigned randomly to IG or CG. Sites randomized to CG will have the opportunity to use Res@t after study recruitment and follow-up are completed.

In addition to introducing the study in regular appointments, advertisements will be carried out via five health insurance companies and the German Society for Child and Adolescent Psychiatry, Psychosomatics, and Psychotherapy (DGKJP) who support the study. Adolescents and parents who are interested in the study will be referred to participating clinics and practices, an appointment for screening and diagnosis will be made, and they will be informed about study details.

Screening for DMUD will take place for all patients within regular child and adolescent psychiatric diagnostics. If a DMUD is confirmed during clinical examination, adolescents and parents will be informed verbally and in writing about the study purpose and procedure including intervention, assessments, and recruitment-site-associated randomized group allocation. Further information concerns confidentiality and data protection procedures including pseudonymization, anonymous data storage at the study center (German Center for Addiction Research in Childhood and Adolescence, DZSKJ, University Clinic Hamburg-Eppendorf, UKE) for 10 years after study completion, possible advantages and disadvantages of participation, and the option to withdraw from the study at any time and without any given reason. Before study enrolment, adolescents and parents give their informed consent and receive all necessary documents on the study.

Since all participants are patients in regular treatment, they will not be financially compensated. However, patients will be offered a voucher for a large selection of online shops as incentive for full questionnaire completion with graded values according to assessment points.

Patient recruitment (first patient in to last patient out) is planned from January 2024 to May 2025. This includes a recruitment period of 12 months and a treatment and follow-up period of 17 months in total.

### 2.3 Eligibility criteria

Recruitment sites have been selected based on the following criteria:

- They are either approved outpatient departments of clinics for child and adolescent psychiatry and psychotherapy,
- Medical care centers for child and adolescent psychiatry and psychotherapy, or
- Practices for child and adolescent psychiatry and/or psychotherapy listed with the local Association of Statutory Health Insurance Physicians or Chamber of Psychotherapists.
- Local healthcare providing professionals need to be experienced with child and adolescent psychiatry and/or psychotherapy. They will comprise specialists for child and adolescent psychiatry, registrars in training as child and adolescent psychiatrists, psychologists, certified psychotherapists for children and adolescents, or psychotherapists for children and adolescents in training.
- All sites will be supervised by a certified experienced child and adolescent psychiatrist and/or psychotherapists.
- The sites are located in Germany.

Participants will be included in the study if they:

- Are between 10 and 19 years old (WHO definition of adolescence);
- Reach the cutoff values for hazardous or pathological use on the Gaming Disorder, Social Media Use Disorder or Streaming Disorder Scale for Adolescents or Parents (GADIS-A/-P, SOMEDIS-A/-P, STREDIS-A/P);
- Fulfill the ICD-11 criteria of disordered or hazardous gaming, social media use, or streaming based on clinical examination;
- Have sufficient German language skills;
- Give written informed consent (for adolescents <16 years with additional informed consent of legal guardians).

OR

- Are a parent, i.e., caregiver, of a patient fulfilling the criteria above.

Participants will be excluded from the study if they:

- Show symptoms of an acute psychosis;
- Are acute suicidal;
- Are substance intoxicated or fulfill criteria of substance use disorder (alcohol, illegal substances, and non-prescribed medication);
- Show a severe reduction in literacy and/or intelligence;
- Have no access to the Internet or are not able to operate on a smartphone, tablet, or computer.

### 2.4 Sample size calculation

The sample size was computed with the procedure tests for Two Means in a Cluster-Randomized Design in PASS 16.0.4 (NCSS, LLC. Kaysville, Utah, United States). A small standardized mean difference (Cohen’s *d* = 0.2) between IG and CG in symptom reduction measured as change from baseline to post-intervention is assumed. The number of study centers is assumed to be 50, each recruiting 27 patients on average. This leads to a feasible sample size of 667 per group. The coefficient of variation of the cluster sizes is set to 0.4 because recruitment is assumed to be heterogeneous due to the different sizes of the study sites. The intra-cluster correlation (ICC) is set to 0.005. With the achievable sample size of 667 patients with respective parents in each of the two groups (*N*_dyads_ = 667) and a two-sided type I error of 5%, the power will be 77%. A dropout rate of 40% before post-intervention assessment (10 weeks) and 20% before follow-up (20 weeks) is assumed. The sample size calculation refers to the three primary hypotheses, taking into account the hierarchical structure. A drop-out rate of 40% before post-intervention assessment (week 10) is considered. With an estimated proportion of adolescents in the outpatient setting with hazardous or disordered digital media use of 20% and a participation rate of 50%, *N* = 13,340 patients must be initially screened. Due to the novelty of the study, conservative estimates were chosen for the assumed effect size and drop-out and participation rates. Digital CBT-based intervention studies with comparable adolescent age groups reported small to medium effect sizes regarding the reduction in depression and anxiety symptoms (36, 37).

Three nuisance parameters (drop-out rate, coefficient of variation, and intraclass correlation coefficient) enter the sample size planning, the assumptions of which are not known with certainty. Therefore, a blinded interim analysis will be performed after the collection of post-intervention data from half of the planned patients (200 per group, 400 total) to estimate the nuisance parameters and possibly adjust the sample size.

### 2.5 Randomization

A stratified cluster randomization will be employed, resulting in each recruitment site (outpatient department or practice) being randomly allocated to the intervention or waitlist control. Stratification is performed according to recruitment site size (annual patient number ≤ 600, > 600 and ≤ 1,600, and > 1,600) and location (urban or rural). An independent statistician at the Institute of Medical Biometry and Epidemiology (IMBE) at the University Medicine Hamburg-Eppendorf will randomly allocate recruitment sites using computerized random number generations within each stratum to IG or CG based on internal standard operating procedures, without knowledge of the identity of recruitment sites (with variable block length). Cluster randomization will be applied to avoid contamination between IG and CG conditions regarding TAU. Based on the resulting allocation, the intervention will be made available immediately (IG) or after the recruitment phase and follow-up data collection is completed (CG).

### 2.6 Assessment and data collection

**Figure 1:**
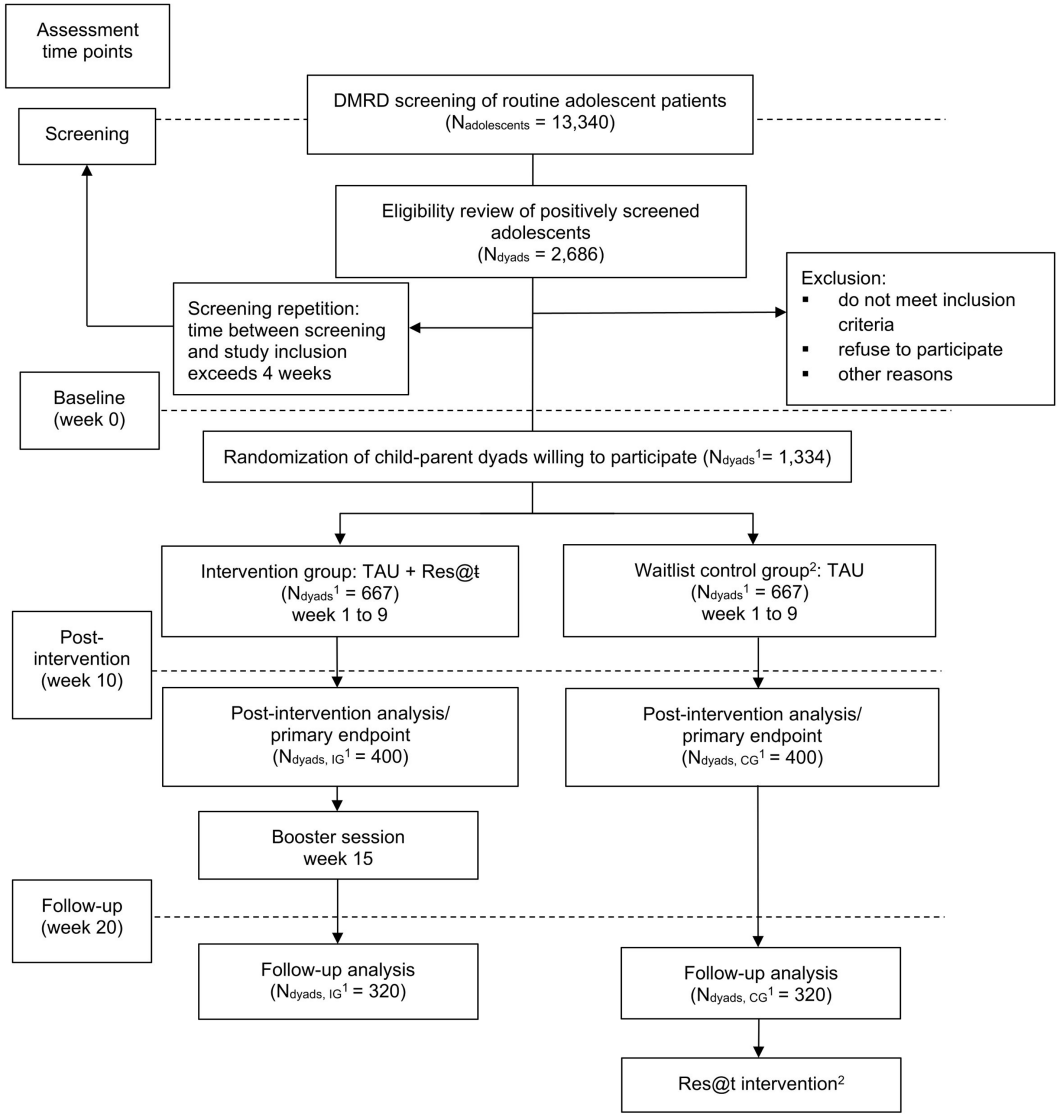
Flow chart of the study. ^1^Total number of adolescent–parent dyads; ^2^Participants of the waitlist control group will be offered the Res@t intervention after data collection is completed. DMUD, digital-media use disorders; TAU, treatment as usual; IG, intervention group; CG, control group.

Clinical assessments with the adolescents and respective parents will be realized at screening, baseline, week 10 (post-intervention), and week 20 (follow-up). By the latter, the durability of the treatment effect should be estimated. Screening and baseline visits should not be apart by more than 4 weeks, otherwise a clinical reevaluation will become necessary. In addition to clinical diagnosis and post-treatment evaluation, all assessments are performed digitally via standardized questionnaires, which are provided on an online platform without the involvement of the clinician. They will be automatically presented to the participants at the time points according to the study protocol. Participants will receive e-mail reminders just before and during the scheduled time frame. All data will be initially stored in a patient dossier on the ISO-certified e-mental health platform run by Embloom^1^ before pseudonymization and secure transfer to the central study center. Regular visits with the clinician will take place within TAU. These should support compliance and adherence to keep the drop-out rate as low as possible. Figure 1 shows all study phases including assessment time points. Table 1 shows all questionnaires and items used in the current study. Table 2 shows the schedule of the questionnaire application.

**Table 1:** Overview of study questionnaires for adolescents and their parents.

	Instrument	Construct	Number of items	References
*Adolescents*				
	Sociodemographics	Sociodemographic data including age, sex, and educational status	18 Items	Standard, adapted to setting
	Media use	Media use (duration and frequency)	13 Items	(38)
	Gaming Disorder Scale for Adolescents (GADIS-A)	Gaming disorder according to ICD-11 criteria	10 Items	(39)
	Social Media Disorder Scale for Adolescents (SOMEDIS-A)	Social media disorder according to ICD-11 criteria	10 Items	(40)
	Streaming Disorder Scale for Adolescents (STREDIS-A)	Streaming disorder according to ICD-11 criteria	10 Items	(15)
	Strength and Difficulties Questionnaire (SDQ)	Emotional and behavioral problems in children and adolescents (self-report)	25 Items	(39, 41, 42)
	Pittsburgh Sleep Quality Index (PSQI)	Sleep quality	24 Items	(43)
	Epworth Sleepiness Scale - Children and Adolescents (ESS-CHAD)	Day-time sleepiness	8 Items	(44–46)
	Perceived Stress Scale (PSS-10)	Psychological stress perception	10 Items	(47, 48)
	Family APGAR	Family functioning	5 Items	(49, 50)
	Family Communication Scale (FCS)	Family communication	10 Items	(51, 52)
	Mindfulness Attention Awareness Scale (MAAS-5)	Mindfulness	5 Items	(53, 54)
*Parents*				
	Sociodemographics	Sociodemographic data including age, sex and gender, parental, educational/ socioeconomic status	18 Items	Standard, adapted to setting
	Parental media use	Parental media use (duration and frequency)	6 Items	(38)
	Gaming Disorder Scale for Parents (GADIS-P)	Gaming disorder according to ICD-11 criteria	10 Items	(43)
	Social Media Disorder Scale for Parents (SOMEDIS-P)	Social media disorder according to ICD-11 criteria	10 items	(55)
	Streaming Disorder Scale for Parents (STREDIS-P)	Streaming disorder according to ICD-11 criteria	10 Items	(12)
	Strength and Difficulties Questionnaire, External Assessment (SDQ-f)	Emotional and behavioral problems in children and adolescents (parental report)	25 Items	(39, 41, 42)
	Media Rules	Media rules	6 Items	(38)
	Family APGAR	Family functioning	5 Items	(49, 50)
	Family Communication Scale (FCS)	Family communication	10 Items	(51, 56)
	Patient Health Questionnaire-9 (PHQ-9)	Parental depression	9 Items	(57–59)
	Generalized Anxiety Disorder-7 (GAD-7)	General anxiety of parents	7 Items	(58, 60)
	Parenting Inventory – Revised (EEI-R)	Parenting style (without subscale about religion)	44 Items	(61)
	Questionnaire on Self-Efficacy in Parenting (FSW)	Parental self-efficacy	9 Items	(62)
	Perceived Stress Scale (PSS-10)	Psychological stress perception	10 Items	(47, 48)
	Ulm Quality of Life Inventory for Parents of Chronically Ill Children (ULQIE)	Quality of life of parents	29 Items	(63)
	Mindfulness Attention Awareness Scale (MAAS-5)	Parental mindfulness	5 Items	(53, 54)

**Table 2:** Diagram of trial activities and measurement time points.

	Study period								
	Enrollment			Intervention		Post-intervention and follow-up		Post-data collection	
Timepoint/Content	Screening (<4 weeks before baseline)	Baseline assessment (week 0)	Allocation	Res@t intervention (week 1–9 and week 15 [booster])	TAU	Post-intervention (week 10)	Follow-up (week 20)	Res@t intervention	TAU
*Enrollment*									
Eligibility screening	X								
Clinical assessment	X								
Informed consent	X								
Randomization IG/CG			X						
*Interventions*									
Intervention group (adolescent-parent dyads)				X	X				X
Waitlist control group (adolescent-parent dyads)					X			X	X
*Assessments*									
Adolescents						IG/CG	IG/CG		
Sociodemographics	X	X							
Media use	X								
GADIS-A	X					X	X		
SOMEDIS-A	X					X	X		
STREDIS-A	X					X	X		
SDQ	X					X	X		
PSQI		X				X	X		
ESS-CHAD		X				X	X		
PSS-10		X				X	X		
Family APGAR		X				X	X		
FCS		X				X	X		
MAAS-5		X				X	X		
Parents						IG/CG	IG/CG		
Sociodemographics		X							
Parental media use		X							
GADIS-P	X					X	X		
SOMEDIS-P	X					X	X		
STREDIS-P	X					X	X		
SDQ-f	X								
Media Rules		X				X	X		
Family APGAR		X				X	X		
FCS		X				X	X		
PHQ-9		X							
GAD-7		X							
EEI-R		X							
FSW		X				X	X		
PSS-10		X				X	X		
ULQIE		X				X	X		
MAAS-5		X				X	X		

GADIS-A, Gaming Disorder Scale for Adolescents; SOMEDIS-A, Social Media Disorder Scale for Adolescents; STREDIS-A, Streaming Disorder Scale for Adolescents; SDQ, Strength and Difficulties Questionnaire; PSQI, Pittsburgh Sleep Quality Index; ESS-CHAD, Epworth Sleepiness Scale - Children and Adolescents; PSS-10, Perceived Stress Scale; Family APRGAR, Family Functionality; FCS, Family Communication Scale; MAAS-5, Mindfulness Attention Awareness Scale; GADIS-P, Gaming Disorder Scale for Parents; SOMEDIS-P, Social Media Disorder Scale for Parents; STREDIS-P, Streaming Disorder Scale for Parents; SDQ-f, Strength and Difficulties Questionnaire – External Assessment; PHQ-9, Patient Health Questionnaire; GAD-7, Generalized Anxiety Disorder Questionnaire; EEI-R, Parenting Inventory - Revised; FSW, Parental Self-Efficacy; ULQIE, Ulm Quality of Life Inventory for Parents of Chronically Ill Children.

### 2.7 Blinding

Practitioners will be informed whether their recruitment site has been assigned to IG or CG at the beginning of the study. Once a patient has been identified to be eligible for study participation and the family gave informed consent to participate, the practitioner will individually inform about the assignment to IG or CG. Evaluating data analysists at IMBE will be blinded and will receive blinded datasets only.

### 2.8 Compliance

Compliance and adherence will be fostered by the treating clinician who observes the participants regularly within the context of normal treatment. Moreover, automatic reminders will be presented via the online app to the participating adolescents and parents. To all participants in the CG, the Res@t intervention will be offered after the completion of study recruitment. Incentives are planned for participants and clinicians to compensate for additional effort. These measures should increase study motivation and reduce drop-out rates.

### 2.9 Res@t digital intervention

Res@t digital is the app-based translation of a manualized CBT-based treatment package comprising offline programs for adolescents with GD (Res@t–A *offline*) and their parents (Res@t–P *offline* (64)). A detailed description of the original program package and its further development can be found in a recent publication by Paschke et al. (22). For better readability, a short summary is given by:

Res@t was created within the theoretical framework of the Trias model of addiction (20) based on clinical experiences and up-to-date research findings on the etiology of DMUD and potentially effective treatment components. During the developmental process, qualitative interviews and focus groups had been conducted with clinical experts, affected adolescents, and their parents on needs and program requirements (26, 64). The findings were considered for the final Res@t version. Original content was adapted to fit digital demands, and specific elements for the treatment of SMUD and VSD have been added. Res@t digital is comprised of nine weekly sessions and one booster session in week 15 of approximately 20 min processing time each. Additionally, participants are encouraged to complete a daily calendar on usage times, non-digital activities, mood, and sleep.

Table 3 gives an overview of the Res@t session contents as offered to adolescents and parents. Modules with adaption to address the type of DMUD are indicated.

**Table 3:** Intervention modules^1^ in Res@t app training for adolescents and their parents.

Session (week)	Adolescents	Parents
1 (1)	Training start The first session presents the purposes and contents of the training and introduces the recurring exercises of self-monitoring and practicing mindfulness. At last, participants are asked to set up training goals, as well as a reward plan together with their parents.	Training start The first session presents the purposes and contents of the training and introduces the recurring exercises of self-monitoring and practicing mindfulness. At last, parents are asked to set up training goals, as well as a reward plan together with their child.
2 (2)^2^	Psychoeducation I^*^ Participants receive a normative comparison during a quiz on digital media usage patterns. They distinguish problematic from unproblematic behaviors in a swiping task and explore the (dis-)advantages of media use, using a four-field-table. Thus, motivation of change should be fostered.	Psychoeducation I^*^ Parents also receive a normative comparison on digital media usage patterns and distinguish problematic from unproblematic behaviors. Then, they get introduced to the assessment of disordered media use and are guided to take their child’s perspective on media use.
3 (3)	Psychoeducation II^*^ The vicious circle of addicted media use is presented and the participants create their personal explanatory model. Furthermore, they learn about the concept of self-control and assign self-control strategies to brief descriptions of distressed adolescents.	Psychoeducation II^*^ The vicious circle of addicted media use is presented to the parents and they learn about different influencing factors, before creating an individual explanatory model. Lastly, they also practice assigning self-control strategies.
4 (4)	Health and sleep hygiene Participants are quizzed about general health knowledge. They analyze their sleep quality, and receive psychoeducation on healthy sleep hygiene. Lastly, participants reflect on sleep hygiene strategies.	Communication Parents are introduced to the concepts of validation and I-messages and test their newly learned knowledge in playful exercises. Lastly, they are asked to practice in their day-to-day lives.
5 (5)	Self-care^*^ Participants are presented with (dys-) functional beliefs about media use and practice cognitive restructuring using so-called “firewall questions.” They explore their talents during a self-interview, and find alternative activities with the help of a decision tree.	Developmental tasks & parenting styles Parents get familiarized to the concepts of developmental tasks and are guided to distinguish the (dis-)advantages of different parenting styles. Lastly, they reflect on their personal situation.
6 (6)	Dealing with Emotions Participants are introduced to the ABC-model of Albert Ellis and practice building “emotional circuits” in an exercise. Furthermore, participants learn more about dealing with emotional distress using skills. Lastly, participants create their individual skills list.	Implementing rules Parents learn about the importance of rules and the differences of rules and requests. The implementation of rules is linked to the use of positive reinforcements, and parents reflect on their individual family rules.
7 (7):	Social relationships^*^ Participants are asked to differentiate the (dis-)advantages of real-life- compared to virtual (para-social) relationships. They are guided to build their individual social network and learn more about techniques to improve their real-life-relationships in a video.	Applying rules Parents explore the (dis-)advantages of rules and reflect on possible changes in their parenting. Furthermore, they are asked to conduct a “family council meeting” with their family.
8 (8)	Communication Participants are introduced to the concepts of validation and I-messages, before assembling the TOP-5 rules against bullying.	Family health Parents learn about stress perception and the influence of parental stress on family health. Also, they reflect on the different roles they exert in their lives and collect ideas on how to deal with stress.
9 (9)	Relapse Prevention^*^ Participants explore individual risk situations as well as warning signs of a possible relapse. Then, they are guided to create their personal list of self-control strategies.	Relapse Prevention^*^ Parents are guided to establish daily routines and alternative activities with their child. Then, they explore risk situations and build plans in case of a relapse. Lastly, parents explore their children’s talents to strengthen their positive mindset.
10 (15)	Booster Participants reflect on their progress and receive a recap-quiz on the contents of the training. Furthermore, they are re-introduced to the different exits of vicious circle of addicted media use. Lastly, participants evaluate their goal attainment.	Booster Parents reflect on their progress and receive a recap-quiz on the contents of the training. Furthermore, they are re-introduced to the different exits of vicious circle of addicted media use. Lastly, parents evaluate their goal attainment.

^1^Content is presented within four tasks per session (i.e., quizzes, psychoeducative videos, fill-in sections for personalized editing). After the first (introductory) session, each session follows the same structure. ^2^Starting with session two, participants are presented with a weekly summary of their calendar entries, self-reflect on their progress during the training, and practice mindfulness with the help of a video instruction at the beginning of each session. ^*^Content is targeted according to the focus on digital games/social media/streaming services.

**Figure 2:**
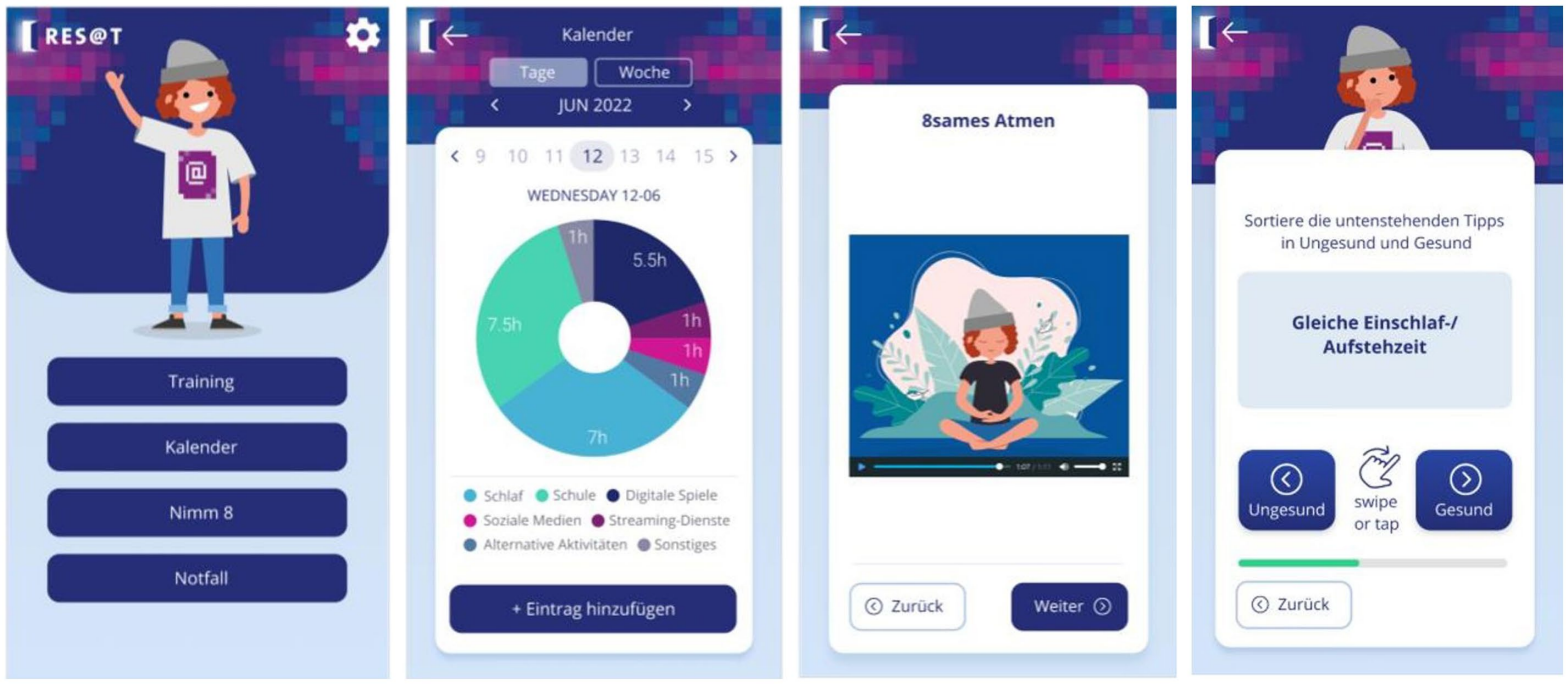
Res@t digital dashboard. The dashboard of the German Res@t application includes an overview of weekly sessions and their completion. It organizes access to the diary, individual sessions, mindfulness exercises, and an emergency button with skills for acute stress reduction and contact numbers of the treating child and adolescent psychiatrist and psychotherapist as well as local emergency departments (left frame). The middle-left frame represents the graphical feedback of diary entries on the duration of digital media use, sleep, and other activities. A mindfulness exercise is shown in the middle right frame. The right frame depicts an example of a swiping task within a quest.

The digital application in the adolescent and the parental versions will be made available via app stores. It is hosted in the secure Embloom platform environment. Based on the study arm allocation, the app, i.e., the intervention content, will be made available directly (for IG) or after individual data collection is completed (for CG). Figure 2 shows examples of the Res@t digital interface (in German language).

### 2.10 Outcome measures

#### 2.10.1 Primary outcome

The primary outcome is the reduction of specific DMUD (GD, PSMU, or VSD) symptoms assessed by the DMUD test battery for adolescents and parents based on differential scores in pre–post comparison.

It includes the Gaming Disorder Scale for Adolescents/Parents (GADIS-A/-P), the Social Media Disorder Scale for Adolescents/Parents (SOMEDIS-A/-P), and the Streaming Disorder Scale for Adolescents/Parents (STREDIS-A/-P). All 10-item scales are identical except for the initial explanation and continuous naming of the specific digital medium of interest (digital games, social media platforms, and video-streaming services) and address either the potentially affected adolescent (self-rating version) or the respective parent (external-rating version). The whole test battery is based on the ICD-11 criteria of GD and has been adapted for SMUD and VSD accordingly. It has been validated in large nationally representative adolescent–parent samples and showed profound psychometrical properties with good to excellent internal consistency, criterion validity, and discriminatory power (12, 15, 40, 55, 65, 66). Accordance between self-rating and parental rating was moderate. Moreover, GADIS-A has been evaluated with Persian and Russian adolescents with good to very good internal consistency and adequate to very good retest reliability (67, 68). Adolescents and parents indicate their agreement with nine statements on a five-point Likert scale (strongly disagree [0]–strongly agree [4]) under consideration of the past 12 months with higher total scale scores resembling more problems. The 10th item reflects the frequency of problems, conflicts, or difficulties due to the digital medium and is answered by choosing one out of four response options (not at all [0]–nearly daily [3]). The questionnaires can be used for a categorical assessment of the DMUD. Accordingly, a score of ≥2 is considered significant regarding the ICD-11 criteria. Moreover, factor analyses confirmed two underlying scale factors, one for *cognitive-behavioral symptoms* (CBS) of problematic media usage and one for *negative consequences* (NC) of the media usage pattern. Cutoffs between scales are comparable, although slightly differing. For GADIS-A and GADIS-P, these are >9 (CBS) and > 5 (NC), for SOMEDIS-A and SOMEDIS-*p* > 8 (CBS) and > 6 (NC), and for STREDIS-A and SOMEDIS-*p* > 6 (CBS) and > 11 (NC).

Children and adolescents will be classified as disordered if the cutoffs for both factors are reached and the time criterion is met. Reaching the cutoff of CBS only suggests hazardous media use. In the clinical context, the DMUD test battery has been regularly used and could prove to be helpful in assessing potential symptom changes during therapy.

Additionally, each treating clinician will assess symptom expression, rate fulfillment of the ICD-11 criteria (based on criteria of GD), and diagnosis of a potential specific DMUD. They will be provided with a digital criterion checklist including examples to enhance interpretation based on ICD-11 suggestions, the clinical interview guideline of the Gaming Disorder and Hazardous Gaming Scale (GDHGS) (69), and clinical expert experience.

In the case of the diagnosis of more than one DMUD, the clinically most prominent one (i.e., by total rating scores and clinical evaluation) will be defined as the main DMUD. This will be addressed during intervention and used as a primary outcome, which was assessed during enrollment, at post-intervention, and follow-up. The differences between screening and post-intervention total scale scores will be investigated primarily.

#### 2.10.2 Secondary outcomes

Secondary outcomes can be divided into psychological, media-associated, parental, and family-associated factors and are assessed with the participating adolescents and parents separately (Table 1). For adolescents, these include adolescent emotional and behavioral problems (based on self-rating and parental rating), sleep-associated aspects, mindfulness, and psychological stress perception, media usage times, and family communication/functioning. For the parents, these comprise mindfulness, quality of life, parental media usage, media rules, parental self-efficacy, and family communication/functioning.

#### 2.10.3 Additional variables

Additional variables that will be included as covariates cover sociodemographic information (age, sex/gender, region of living, and parental status), parental mental distress, and parenting style (Table 1). Moreover, data on media usage and app usage patterns, i.e., media usage time as well as number of app usage sessions, days, weeks, quests started, quests completed, mindfulness exercises observed, and calendar entries, as well as the relative completion of modules and complete training will be collected.

### 2.11 Data collection and management

All data will be collected on an ongoing basis via the Res@t application and stored within the ISO-certified Embloom platform on secure servers with regular backups. Data will be pseudonymized before transferring it in an encrypted form to the secure UKE server. Sensitive participant data will be accessible only to the treating practitioner within individual digital patient folders (within the recruitment center) and authorized admins of Embloom (across the recruitment centers). The latter will have the primary responsibility for verifying the integrity of the database and securing pseudonymized data transfer. A statistician at the UKE who is independent of the evaluation team will monitor trial conduct and data collection manually in addition to automatic monitoring of data completeness by the software. Moreover, pseudonymized data will be reprocessed to fit all structural requirements for the analyses. The principal study center at the DZSKJ will be responsible for managing and archiving the transferred database after analysis. All participant data will be always handled confidentially and in accordance with the General Data Protection Regulation (GDPR).

### 2.12 Data analysis

Data will be analyzed by the independent statisticians at the IMBE with sophisticated experience in multicenter therapy studies. Descriptive statistics will be presented with respect to both the entire sample and group-wise. The primary analysis is based on the intention-to-treat (ITT) approach with all randomized patients. The significance level will be set at 5% (two-sided). All three primary hypotheses are structured hierarchically. The second primary hypothesis will only be evaluated in a confirmatory way if the first primary hypothesis leads to a significant test result. The third primary hypothesis will only be evaluated in a confirmatory way if the second primary hypothesis leads to a significant test result. For the analysis of each primary hypothesis, a linear mixed model with implicit maximum likelihood correction of missing values under the missing-at-random assumption will be calculated, respectively. The change in DMUD symptoms from baseline to post-treatment will be used as an outcome variable, randomization group, type of DMUD (social media, gaming, streaming), recruitment site size and location as fixed effects, individual recruitment site and patient nested within site as random effects, and the baseline DMUD score as a covariate. The interaction between the randomization group and media usage pattern (hazardous vs. pathological) as well as effects of incomplete dyads will be tested within secondary analyses.

To examine the impact of missing values on the outcome of the primary analysis, sensitivity analyses will be performed using multiple imputation methods to replace missing values. The primary endpoint at follow-up and the secondary endpoints will be investigated exploratorily using analogous methods, which are adequate for the scale type. The following subgroup analyses are planned: severity of DMUD, sex, education, and socioeconomic status. Qualitative assessments of treatment provider satisfaction will be supplemented. Details of the analysis will be specified in a statistical analysis plan. The analysis will be performed with the latest version of the software package R (70).

### 2.13 Quality assurance and monitoring

All recruitment sites will be supervised by and stay in close contact with the principal study center at UKE during the complete course of the study.

At the beginning of the recruitment, a Data Safety Monitoring Board (DSMB) will be implemented with meetings during initiation, regular monitoring visits, and after study closeout. The DSMB will be comprised of experienced scientists not otherwise involved in the study. Members will be independent of the investigarors and the trial sponsor and will have no conflicts of interests. The DSMB will frequently oversee trial processes and data collection based on the requirements of Good Clinical Practice. These include monitoring of study and recruitment progress, considering adherence to the inclusion criteria, study procedure, recruitment rate, completeness of study documents, protocol deviations, loss-to-follow-up data, serious adverse events (SAEs), adverse events (AEs), and/or newly emerged evidence of potential harmful effects of the intervention, and all potential study problems. Meetings will be held in closed and open manner. The latter includes the discussion of management issues with the coordinating investigator at the principal study center. Recommendations on study continuation, necessary design changes, or the end of the trial will be given by the DSMB without the involvement of the coordinating investigator.

### 2.14 Safety

Data safety is secured via confirmation with the Information Security Management System Standard (ISO/IEC 27001:2013y) in accordance with the Declaration of Applicability v 2.0 d.d. 22/10/2019. This certificate is valid within the scope of information security related to the development and management of e-health applications for healthcare providers and the purpose of measuring, monitoring, and treating psychological and physical symptoms. Data collection, transfer, and storage are described within a data protection concept, which has been approved by the data protection officer at the UKE.

Participant safety, especially the safety of minor-aged patients, is secured primarily by the clinicians during regular treatment. They will continuously monitor adverse events (AEs) and serious adverse events (SAEs) and report these as an (S)AE comment within the case report form of Embloom software. (S)AEs will be rated based on the NCI Common Terminology Criteria for Adverse Events (71). They will be carefully monitored, documented, and reported to the coordinating investigator and the Data Safety Monitoring Board (DSMB) members within 1 week of the initial observation. DSMB experts will comment on potential causal study relations to identify serious study-related events (SSREs), to determine benefit–risk of trial continuation and adequate participant support. SSREs might include suicidal ideation and behavior, self-harming behavior, worsening of general wellbeing, mental distress, or psychiatric comorbidities with an indication for hospitalization. SAEs and SSREs will be reported to the local ethics committees.

### 2.15 Clinical trial registry

The trial is registered on the German Clinical Trials Register (DRKS00031043, https://drks.de).

## 3 Discussion

Given the rising prevalence of DMUD, especially during the vulnerable period of adolescence, and a severe lack of evidence-based treatment options, especially for this age group and their parents, Res@t digital aims to close a significant gap. Res@t is the first model-based manualized training program to reduce DMUD symptoms that specifically includes adolescents and parents. It has been applied and pilot-tested in different settings and translated into a digitalized version. Within a large clinical consortium, the effectiveness of Res@t digital will be independently evaluated in a multicenter cluster RCT study in the clinical context. This setting was chosen to investigate a novel intervention for those in urgent need and secure safety of mostly minor patients. In the case of a positive evaluation, Res@t digital can be applied quickly and support potentially limited local medical care structures by providing evidence-based treatment content. The reliable screening for and treatment of DMUD can prevent aggravation and chronification of symptoms. Res@t digital might be used to bridge waiting time and support patients who initially avoid face-to-face interventions due to factors such as lack of motivation, logistic, time, or financial problems. Accordingly, a recent systematic review and meta-analysis could not find differences between the treatment effectiveness of face-to-face intervention and digital intervention in the context of anxiety disorders (72).

In addition, applying the Res@t app with its up-to-date treatment material within a blended therapy context might enrich and facilitate face-to-face interventions and allow multiple self-guided repetitions to consolidate content. Reviews suggest positive effects of such an approach across age groups including adolescent patients (73, 74) with evidence of beneficial effects of guided (versus unguided) digital interventions (75). Moreover, the beneficial effects of additional face-to-face interventions are assumed to vary according to the reachability of adolescents with DMUD. Therefore, within a subproject of the Res@t digital consortium, it will be investigated how much face-to-face contact is required to guide affected adolescents through the Res@t digital intervention. These adolescents will be supported by the youth welfare system but will not yet be involved in outpatient care.

The Res@t application ensures highest quality standards and has the potential to reduce health care costs. As with other e-health interventions, it might help reaching a broader patient group (76), not only for affected German adolescents but potentially worldwide. By involving primary healthcare providers in the evaluation process, this study is settled within direct healthcare research, mirroring immediate realities of life and high practical relevance.

Based on our current knowledge, Res@t digital has the potential of an effective, safe, and cost-efficient treatment option for a significant number of adolescent patients and their parents. In the future, new modules could be added or current modules could be altered and tested to fit the demands of different settings, such as schools, youth, and counseling services, or expand to additional age groups such as young adults or younger children.

## 4 Limitations

A cluster-randomized design was chosen to exclude contamination effects on TAU within IG and CG. However, by involving practitioners actively in the study and providing structured diagnostic tools for the assessment of DMUD, it cannot be ruled out that TAU is altered by an increased awareness of the clinicians. This effect might be stronger in recruitment sites that are allocated to CG as child, and adolescent psychiatrists and psychotherapists strive to address detected problems, where no immediate additional support is available. More attention to the CG compared with the IG would result in a potential reduction in the overall outcome effect. Measures taken against this will include proper instruction of recruiting practitioners. Moreover, the cluster-randomized design does not allow initial sample stratification, e.g., regarding the type of DMUD, sex, age, and single versus dyad study participation. However, the number of recruitment sites is aimed to be so high that these variables should be distributed randomly. Another potential drawback of cluster randomization is that the waiting CG will get access to the intervention after their individual data collection but not after full data collection. Based on the feedback from recruiting clinicians, waiting times of several months up to a year in that case would not have been acceptable. Clinicians will be instructed to not to alter TAU based on their experiences with Res@t digital. However, contamination effects on TAU cannot be ruled out.

A potential risk for project realization could be that recruiting a significant number of parent–child dyads are more challenging than expected. Therefore, a large clinical consortium has been established. The heterogeneity of the consortium might induce additional variance (TAU, support of study). However, measures are being taken to control this effect. These include clear recruitment instructions, attractive remuneration for successful recruiting, including recruitment site size into the stratification process of randomization and recruitment site itself as a nested factor into the multilevel model analyses. Participant dropout has been estimated conservatively but is especially challenging since parent–child dyads are addressed. Hence, dropout could be higher than expected. Rates should be kept as low as possible by personal contacts and motivation via practitioner, reminders, app visualization of progress, fostering parent–child interaction during the intervention process, and family incentives in the case of full assessment completion. Moreover, separate analyses will be performed on incomplete dyads.

The evaluation of the primary outcome includes self-rating, parental rating, and expert rating in a hierarchical order. The use of different ratings for diagnosis and monitoring is highly common in clinical practice. On the one hand, it can be observed that affected adolescents might show a tendency to understate present symptoms due to feelings of shame and social desirability (77), lowered introspective abilities (78), self-regulatory and executive control functions (79), or denial and concealment typical for addictive disorders (80). On the other hand, parents’ worries often lead to a focus on (potentially) negative consequences of problematic behavioral patterns of their children. This could result in a more critical symptom evaluation or even exaggeration (81–83). The clinician tries to integrate all available information and applies diagnostic criteria for evaluation. Consequently, diverging symptom estimates might occur which need to be considered when interpreting the evaluation results.

Moreover, since the aim is a large sample, no objective measures can be applied to evaluate intervention effectiveness for the complete sample, even though these would be highly appreciated. Hence, a second subproject within the Res@t consortium focusing on DMUD-associated sleep–wake alterations will apply neuropsychological and physiological measures including actigraphy in a subsample.

## 5 Conclusion

Res@t digital is the first model-based and app-based intervention that addresses the vulnerable group of adolescents with DMUD or hazardous use patterns and a respective parent in order to reduce symptoms and negative sequelae, prevent chronification, and foster the mastering of developmental tasks. After standardized diagnosis, treatment effectiveness will be evaluated by applying a multicenter cluster-randomized pre–post follow-up waitlist control group design with evaluator blinding within the primary clinical care setting. It will be tested if Res@t digital is feasible for and effective in outpatients who receive the intervention in addition to TAU compared with the control group with TAU only. By selecting a study setting within primary clinical care, optimal patient security can be assured, blended-therapy approaches are made possible, and important implementation factors can be accounted for to increase acceptance and accessibility from the very beginning. If effectiveness is shown, Res@t will be made available for long-term perpetuation, considering practicability and potential independence of local care structures and cost-effectiveness.

## Ethics statement

The study was approved by the Ethics Committee of the Medical Board Hamburg. The study will be conducted in accordance with the local legislation and institutional requirements. Written informed consent for participation in this study will be provided by the participants and their legal guardians/next of kin.

## Author contributions

KP and RT acquired the funding for the trial. KP contributed to the conceptualization, intervention content, study design and methodology, and prepared the original draft. AZ provided guidance on the study design and statistical issues. KB contributed to manuscript preparation including editing and visualization. SD and TK contributed to project implementation. SD, NA, AP-K, and OR served as scientific advisors. J-OC and A-LS contributed to the intervention content. RT contributed to the resources and supervision. All authors contributed to the article and approved the submitted version.

## Footnotes

^1^ https://www.embloom.com
